# Optimization of sampling and monitoring of vegetative flushing in citrus orchards

**DOI:** 10.1371/journal.pone.0233014

**Published:** 2020-05-20

**Authors:** Everton Vieira de Carvalho, Juan Camilo Cifuentes-Arenas, Carlos Augusto Santos de Jesus, Eduardo Sanches Stuchi, Silvio Aparecido Lopes, Eduardo Augusto Girardi

**Affiliations:** 1 Departamento de Ciências Agrárias, Ambientais e Biológicas, Universidade Federal do Recôncavo da Bahia, Cruz das Almas, Bahia, Brazil; 2 Departamento de Fitossanidade, Faculdade de Ciências Agrárias e Veterinárias, Universidade Estadual Paulista “Júlio de Mesquita Filho, Jaboticabal, São Paulo, Brazil; 3 Fundo de Defesa da Citricultura (Fundecitrus), Araraquara, São Paulo, Brazil; 4 Embrapa Mandioca e Fruticultura, Cruz das Almas, Bahia, Brazil; 5 Estação Experimental de Citricultura de Bebedouro, Bebedouro, São Paulo, Brazil; Instituto de Biologia Molecular y Celular de Plantas, SPAIN

## Abstract

Citrus trees produce flushes throughout the year, but there are no criteria established for a precise shoot monitoring in orchards under tropical climate. Methods for quantification of flush dynamics would be useful for horticultural and pest management studies because different insect vectors feed and reproduce on flushes. We estimated the minimum number and distribution of trees for sampling and determined the flushing pattern over time in ‘Valencia Late’ orange trees grafted onto ‘Swingle’ citrumelo rootstock. Shoots within a square frame (0.25 m^2^) on two sides of the canopy were counted and classified by their phenological stage. The minimum number of samples was estimated using the mean number of shoots and area under the flush shoot dynamics (AUFSD). The temporal and spatial distribution analysis was performed by Taylor’s power law and by multiple correspondence analysis (MCA). Additionally, a shoot maturity index (SMI) based on visual qualitative assessment of flushes is proposed. Considering the mean number of shoots, it was necessary to sample two sides of 16 trees to reach a relative sampling error (*E*_*r*_) of 25%, whereas by the AUFSD, only five trees were necessary to reach an *E*_*r*_ of 10%. Flushes were predominantly randomly distributed over time and space. Testing eight transects, sampled trees should be distributed throughout the block, avoiding sampling concentration in a certain area. MCA showed that the west side and the upper sampling positions of trees were more likely to be associated with younger shoots. AUFSD and the evaluation of both sides of the canopy yielded a smaller number of trees to be assessed. The SMI was a reliable metric to estimate the shoot phenology of orange trees, and correlated well (*R*^*2*^ > 70%) with the mean number of shoots within the square frame. Therefore, SMI has the potential to make shoot monitoring in the field more practical.

## Introduction

Citrus is among the most important fruit crops in the world and is cultivated under a wide range of climate types [1]. Vegetative growth in citrus trees is affected by environmental factors and occurs in several cycles (flushes) over the year. It is well documented that under Mediterranean and temperate climatic conditions, flushes emerge in well-defined cycles, whereas in tropical and subtropical regions, they appear more unevenly [2]. Estimation of citrus flush patterns can provide useful information for studies on plant physiology and decision making on management of citrus production.

Scouting of flushes may also contribute to integrated pest management decisions, since new shoots are one of the main entry points of vector-borne pathogens of citrus. For instance, citrus variegated chlorosis (CVC) is caused by *Xylella fastidiosa* and vectored by several leafhoppers [3]. Citrus canker is caused by *Xanthomonas citri* subsp. *citri*, whose incidence and severity are exacerbated by the damage produced by the citrus leafminer (*Phyllocnistis citrella* Stainton) [4]. Finally, huanglongbing (HLB), which is considered the most damaging citrus disease, is associated with *Candidatus* Liberibacter spp. bacteria disseminated by the Asian citrus psyllid (ACP), *Diaphorina citri* Kuwayama and the African citrus psyllid, *Trioza erytreae* (Hemiptera: Liviidae) [5–8]. Therefore, knowledge about the distribution pattern of the young shoots in different parts of the plant and among different plants of the orchard throughout the seasons is useful for deciding how and when to sample flushes [9].

Despite increased interest in shoot monitoring for integrated pest management programs to determine critical periods of host favorability for area-wide control [10,11], there are few standardized recommendations for sampling flushes in citrus orchards [12]. Direct assessments are still the most used methods in the field. These methods include visual inspection of plant organs, or selected branches of standardized size, or square/cubic/circular frames to count flushes, and record the presence or absence, or assign qualitative values to the evaluated traits [9, 13,14].

In citrus crops, a few studies have evaluated shoot growth over time using counting of flushes in selected branches or within frames, but only a small number of trees were sampled, and the focus was mainly on ACP correlations [15–17]. In Florida, USA, Hall and Albrigo [12] used a cubic frame of 3.47 dm^3^ and estimated that sampling 40 trees in a uniform block of < 1 ha would provide a reliable estimate of flush abundance in the orchard. Shoot monitoring using a large number of trees could be a laborious and expensive activity, yet a reliable sample size must be used. In addition, the use of small sampling frames may cause higher variability and underestimation of the abundance of flushes, since the smaller the area, the lower the probability of detecting a flush, hence the greater the number of sampling points that will be required [18].

Given the limited information on monitoring of citrus flushes, especially for tropical conditions, we conducted an experiment to evaluate statistical procedures for estimating the minimum sample size (number of trees), to define a sampling scheme (location of trees within the area) and to determine the temporal and spatial distribution pattern of flush shoots of sweet orange trees. Taken together, this information can be useful for monitoring shoot growth patterns of citrus as a tool to support horticultural studies and integrated pest management programs.

## Material and methods

### Experimental area and plant material

The experiment was carried out in the municipality of Bebedouro, São Paulo State (SPS), Brazil (20°53’16” S, 48°28’11” W), at 600 m a.s.l., with Aw climate, characterized as tropical with dry winter (Köppen climate classification system) [19]. Meteorological conditions were monitored using a CR10 measurement and control system (Campbell Scientific Inc.) located in the area.

The orchard was 1 ha in size, non-irrigated and planted with ‘Valencia Late’ sweet orange [*Citrus sinensis* (L.) Osbeck] (5 years old, ≈ 2.33 m tall) grafted onto ‘Swingle’ citrumelo rootstock [*Citrus paradisi* Macfad. x *Poncirus trifoliata* (L.) Raf.]. Trees were planted at 2.0 m within-row spacing, 6.0 m between-row spacing, 833 trees ha^-1^, with 24° of azimuthal orientation of the rows. The side of the plant facing the N-W or the S-E was named west or east, respectively. During the evaluation period, the orchard was not pruned and received the standard management practices recommended for citrus crops in SPS. Contact insecticides were sprayed every 10 days mainly for ACP control.

A total of 160 trees were selected in four rows (40 trees per row). Only trees with healthy overall appearance (asymptomatic) were used. All trees were individually identified and labeled. The same trees were evaluated in all sampling dates. When HLB symptoms were identified on a tree, it was immediately removed and replaced with a healthy one in the same row.

### Evaluation of plant phenology

Shoot evaluations were carried out every 21 days during a 12 month period. This time interval was chosen because it is shorter than the required period for a complete shoot development cycle (bud swelling to mature shoot) of ‘Valencia Late’ sweet orange (35–45 days at 24–26 ^o^C) [7], which ensured that at least one evaluation would be carried out for all shoot cycles that occurred during the evaluation period.

Two shoot evaluation methods were used. The first method was quantitative. All the shoots located within the projection of a 50 cm x 50 cm square frame placed on the outer surface of the canopy, 1.5 m from the base above ground level, were counted and classified within distinct phenological stages, ranging from vegetative stage v1 (bud swelling) to v7 (fully mature shoot), following the method used by Fundecitrus [20] adapted from Agustí et al. [21]. Assessments were performed on east and west sides of the tree to nullify differences in sun radiation exposure. The reproductive and mixed shoots that were present along with the vegetative shoots during late winter and early spring (main flushing period), were also counted but not included in the analyses because the scale used to classify shoots only considers vegetative ones. This facilitates comparisons between all assessment dates, including when only vegetative flushes were present.

The second method was qualitative. The canopy was divided into eight sampling positions: four on each side, two in the upper (SP1 and SP2) and two in the lower half (SP3 and SP4) of west face of the tree in the planting line; two in the upper (SP5 and SP6) and two in the lower half (SP7 and SP8) for east face (S1 Fig). In each sampling position, the predominant phenological stage of the shoots (> 50% of total shoots in that sampling position) was quantified as scores from 1 to 7 for v1 to v7, respectively. This allowed estimation of the shoot maturity index (SMI), which is the total sum per tree of the scores in each sampling position and ranged from 8 (v1 in all sampling positions) to 56 (v7 in all sampling positions).

### Temporal and spatial analysis of new shoots

We first determined the relationship between variance (*S*^*2*^) and mean $\left(\bar{X})\;(S^{2}={a\bar{x}}^{b}=\beta_{0}x^{\beta_{1}}\right)$, with an approximation of the coefficients obtained by simple linear regression of the logarithmically transformed variance and mean (natural logarithm), where *a* (*β*_*0*_) is the intercept and *b* (*β*_*1*_) is the slope of the regression line. A value of *b* = 1, *b* < 1 or *b* > 1 indicates random, regular or aggregate distribution, respectively, which provides the description of the distribution pattern of the flush shoot population in the orchard [9,22]. To determine whether *β*_*0*_ and *β*_*1*_ ≠ 0 or 1, we tested whether the value belonged to the 95% confidence interval of the parameter (significant if 0 or 1 ∉ ± 95% CI) [23]. For the temporal distribution analysis, the mean number of new shoots of the 160 trees on each sampling date was considered as a data point. The spatial distribution of new shoots was analyzed for each sampling date by estimating the mean and variance values of eight adjacent trees in the row, totaling 20 data points for each assessment date.

### Optimum sample size and sampling scheme

Prior to this analysis, the area under the flush shoot dynamics (AUFSD) curve during the whole period of assessment was calculated for each sampled tree (taken as a replication), as indicated:

$$AUFSD=\sum_{i=1}^{n-1}\left(\frac{{{NS}_{i+1}+NS}_{i}}{2})*(T_{i+1}-T_{i}\right),$$ Eq (1)

where *NS*_*i*_ is the number of new shoots in stages v1 to v7 in the *i*^*th*^ observation and *T*_*i+1*_*-T*_*i*_ is the number of days between observations [24]. Because some of the trees sampled showed HLB symptoms throughout the evaluations and were removed from the orchard, the AUFSD was estimated only for the HLB asymptomatic trees evaluated from the first to the last date. The unit of the AUFSD is ‘new shoots days’ (NS-days). Computing the area curves is a simple method that uses trapezoidal integration (also known as the mid-point rule) to summarize the progress data of a variable of interest, whether it is continuous or discrete [9]. This is a standard method widely used to study disease resistance in plants [25–27], population dynamics of insects [28,29], and cumulative thermal requirements for crop or pest development (with modifications) [30].

The optimum sample size (number of trees to be sampled) was estimated using the data from the AUFSD (Eq 1) as well as the mean number of new shoots within the square frame per tree. For AUFSD, the estimation was performed only once, at the end of the experiment. For the mean number of new shoots, the total number obtained for the 160 trees was always evaluated, including for those that replaced trees removed due HLB disease. Thus, the optimum tree sample size was determined as ${n=[\sigma /\left(E_{r}*\bar{X}\right)]}^{2}$ [9], where *σ* is the standard deviation and *E*_*r*_ is the relative sampling error (*E*_*r*_ = [standard error of the mean]/mean, which is the degree of accuracy required or tolerated), and $\bar{X}$ is the mean. A nonlinear regression (y = a+b/x, selected by comparing its *R*^*2*^ value with other models) of the mean number of new shoots against the optimum sample size was carried out. We varied the value of *E*_*r*_ from 5% to 25% (0.05 to 0.25) to show and discuss several sample sizes according to the tolerance of the error.

Because the trees were not randomly selected, the data were validated by generating 20 sets of non-repeated random numbers with 3, 6, 12, 24, 48 and 96 trees each, among the 160 trees evaluated. The optimum sample size for all the 20 sets was also estimated through nonlinear regression of the relative sampling error (*E*_*r*_) as a function of the sample size [*E*_*r*_ = 1/(*a*+b*$\sqrt{n}$)], to obtain the highest *R*^*2*^ value, where *a* is the intercept and *b* is the slope.

After the optimum sample size was estimated, we designed different paths or sampling schemes (eight transects) running the entire block distributing these points. For each sampling date, we estimated the mean number of new shoots and compared the values with the overall average number of new shoots considering all 160 trees. The eight transects were: I to IV: solid block of 16 adjacent trees in two adjacent rows, in different sectors of the plot (more aggregated schemes); V and VI: all 16 trees selected in two adjacent rows, sampling two trees (one on each row) every five trees in different sectors of the block (“*single 2 by 5*” scheme); VII: two pairs of adjacent rows separated by four non-sampled rows, and on each pair of rows, eight trees were selected in a ‘W’ scheme (“*double 1 by 8*” scheme); and transect VIII: a combination of transects V to VII, selecting two pairs of rows separated by four rows, and sampling two adjacent trees (one in each row) every ten trees, resembling a “Z scheme” (“*double 2 by 10*” scheme). See S1 Appendix for details.

The data on AUFSD and mean number of shoots were analyzed for homoscedasticity and normality by the *F*-test and Shapiro-Wilk test, respectively. To compare the shoot dynamics between the two sides of the canopy, Student's *t*-test for paired samples was applied.

### Distribution of shoots in the canopy

For each sampling date, the association between the predominant phenological stage of the new shoots with the sampling position (upper and lower half) and canopy side was studied using multiple correspondence analysis (MCA), a multivariate statistical technique that aims to study the interdependence of non-metric attributes (three or more categorical variables) and whose main result is a perceptual map that allows observing the proximity between the levels of variables [31]. MCA has been widely used to study spatial patterns of ecological traits [32,33], relationships between functional diversity of microorganisms and cultivated plants [34], and genetic diversity for breeding purposes [35]. Here we evaluated its use as a way to study where and when the new shoots are more likely to be found.

To simplify the analysis, the shoots that were initially classified in phenological stages between v1 and v7 were clustered into groups that indicated a specific developmental phase, as indicated by Cifuentes-Arenas et al. [7]. In this clustering, stages v1 to v3 were grouped as developing shoots (D), whereas v4 to v5 were grouped as developing/maturing shoots (D_M). Finally, the v6 to v7 shoots were classified as mature (M). Similarly, sampling dates were pooled, grouping data of every three assessment dates (1^st^ to 3^rd^, 4^th^ to 6^th^, 7^th^ to 9^th^, 10^th^ to 12^th^, 13^th^ to 15^th^, and 16^th^ to 18^th^), as they share similar climatic characteristics by being in the first or second half of each season and to simplify the visualization and interpretation of the results.

### Shoot maturity index

For each date of assessment, 20 sets of 5, 10, 20, 40 and 80 trees each were randomly selected to study the relationship between the shoot maturity index (SMI) from each side of the tree or from the whole tree and the mean number of new shoots counted within the square frame. Then, for each dataset simple linear regression of the mean number of new shoots counted within the square frame against the SMI was carried out. The optimum sample size with the SMI was analyzed observing the improvement in the coefficient of determination *(R*^*2*^) after increasing the number of trees randomly sampled per dataset.

Statgraphics Centurion XVII (Statpoint Technologies Inc.) was used to perform all analyses. Raw data from the quantitative and qualitative methods are available in S2 Appendix and S3 Appendix.

## Results

### Overall weather conditions

**Fig 1:**
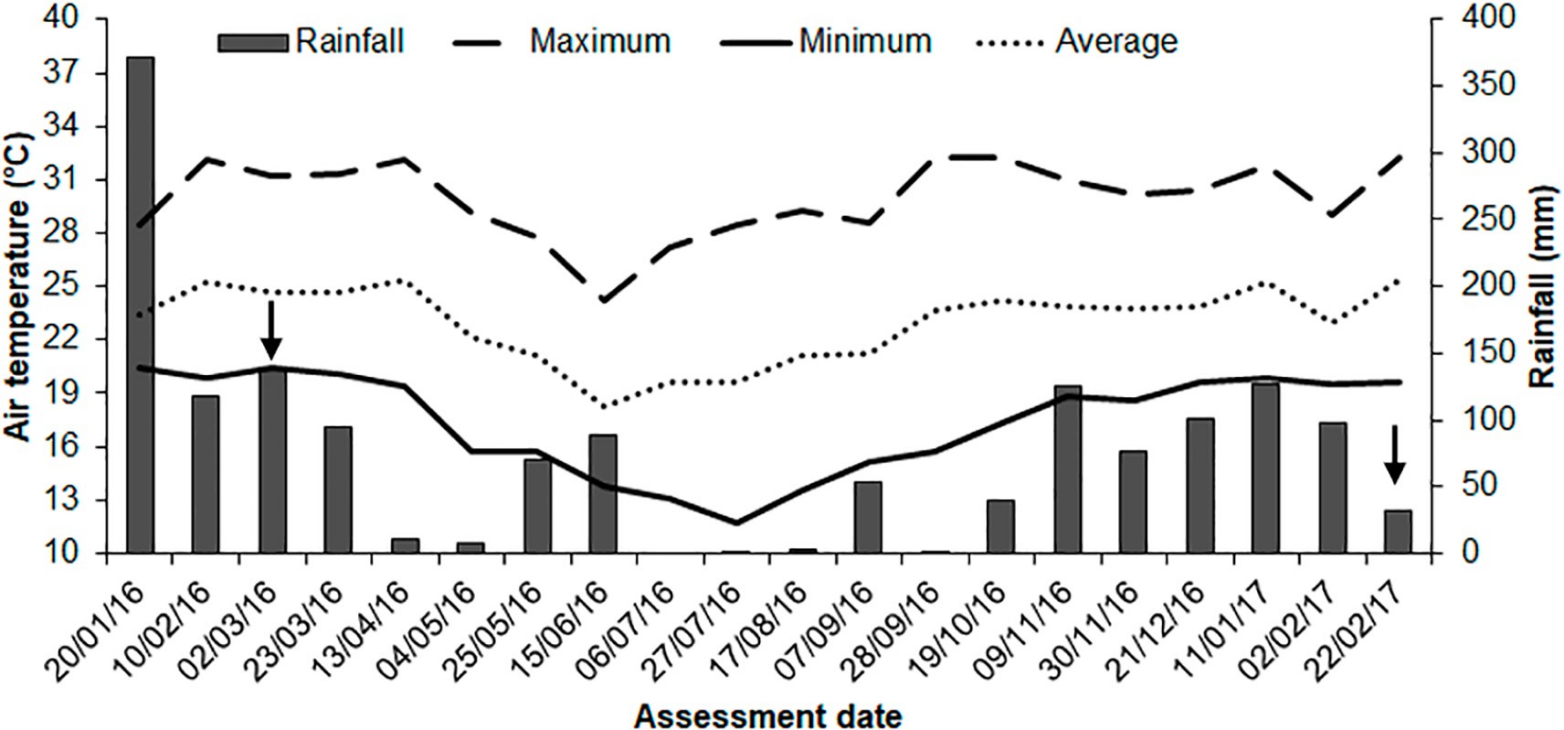
Overall weather conditions during the evaluation period. Cumulative rainfall (mm) and average air temperature (ºC) registered in the periods between sampling dates (each 21 days), from January 2016 to February 2017 in the experimental area. The arrows indicate the first and last assessment dates.

The average air temperature and relative humidity during this study were 22.7°C (avg. max. = 29.8°C, avg. min. = 17.0°C) and 66% (avg. max. = 88%, avg. min. = 37%), and the cumulative rainfall was 1560 mm. The warmest months were March and April 2016 and February 2017, with averages of 24.6°C and 69% of RH, while the coldest were May, June and July 2016, with averages of 19.7°C and 64% of RH (Fig 1). Total radiation at midday in the experimental area varied from 1.91 to 16.23 kJ m^-2^ day^-1^ during the evaluation period.

### Temporal and spatial distribution of new shoots

**Fig 2:**
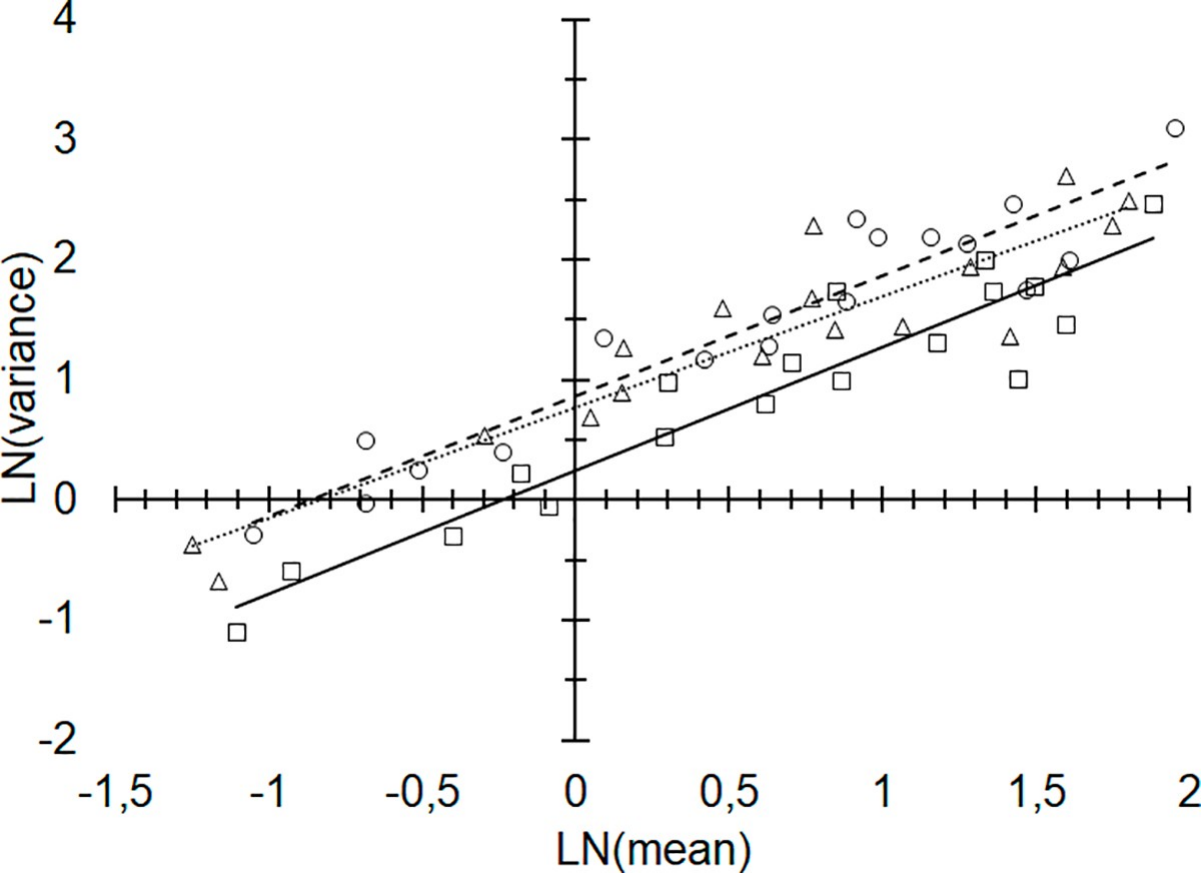
Variance and mean on LN x LN scale. Relationship between mean and variance (log-transformed) of young flush shoots (growth stages v1 to v7) counted within the 0.25 m^2^ square frame projected on two sides in the middle section of the canopy (west side, dotted line and triangles; east side, dashed line and circles; mean from both sides, continuous line and squares). Each point represents the data from the 160 trees for each assessment date.

The simple linear regression of the log-transformed variance and mean indicated that, overall, the variance increased as a function of the mean (Fig 2) during the evaluation period (west side: *F*_*1*, *16*_ = 94.76, *P* < 0.0001, *R*^*2*^ = 0.8555; east side: *F*_*1*, *16*_ = 138.85, *P* < 0.0001, *R*^*2*^ = 0.8967; average of both sides: *F*_*1*, *16*_ = 130.15, *P* < 0.0001, *R*^*2*^ = 0.8905).

Taylor’s power law coefficients (Table 1) indicated that the temporal distribution of the flush shoots during the year of the study was classified as random for both west and east sides, as well as for the average of both sides.

**Table 1:** Coefficients (±SE) of Taylor’s power law from the linear regression of the log-transformed data of variance against the mean for determination of the temporal distribution. Data from the 160 trees on each of the 18 sampling dates.

Parameter	West side			East side			Both sides		
	Value	H_0_: *β* ≠ 0^a^	H_0_: *β* ≠ 1^a^	Value	H_0_: *β* ≠ 0^a^	H_0_: *β* ≠ 1^a^	Value	H_0_: *β* ≠ 0^a^	H_0_: *β* ≠ 1^a^
*β*_*0*_	0.77(0.10)	*	*	0.87(0.09)	*	0.1279	0.25(0.10)	*	*
*β*_*1*_	0.92(0.09)	*	ns	1.00(0.09)	ns	0.3983	1.03(0.09)	*	ns

^a^ statistical significance was determined as 0 or 1 ∉ ±95% CI.

**Fig 3:**
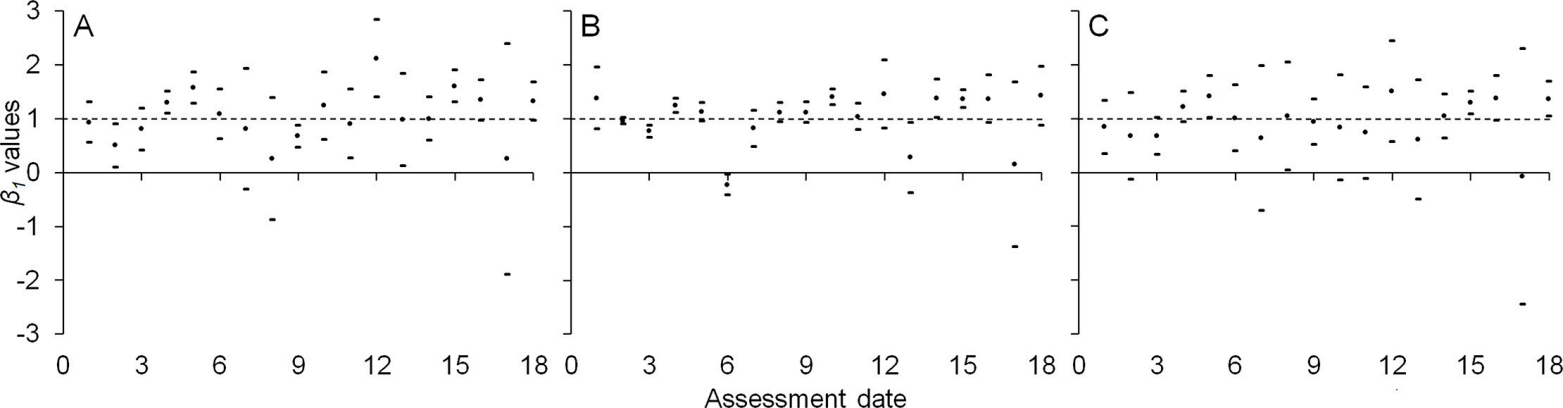
Spatial distribution of new shoots. Values of *β*_*1*_ parameter (points) and 95% confidence intervals (upper and lower dashes for each point) of Taylor’s power law from the linear regression of the log-transformed data of variance against the mean for determination of the spatial distribution on each assessment date (to see the detailed results of the regressions, see S1 Table. Horizontal dotted line indicates *β*_*1*_ = 1.

Similar to the temporal distribution of flush shoots, simple linear regression revealed that the variance tended to increase with the mean value in the spatial distribution analysis (Fig 3A to 3C). The coefficients of Taylor's power law showed that, for the west side (Fig 3A), the distribution of flush shoots was random in most evaluations (1^st^, 2^nd^, 3^rd^, 7^th^, 9^th^, 10^th^, 11^th^, 13^th^, 14^th^, 16^th^, and 18^th^), aggregate in the 4^th^, 5^th^, 12^th^ and 15^th^, and uniform in the 8^th^ and 17^th^ evaluations. On the east side (Fig 3B), the shoot distribution was similar, random in the 1^st^, 2^nd^, 3^rd^, 5^th^, 7^th^, 8^th^, 9^th^, 11^th^, 12^th^, 16^th^ and 18^th^ evaluations, uniform in the 6^th^, 13^th^ and 17^th^, and aggregate in the 4^th^, 10^th^, 14^th^, and 15^th^. When the average from both sides was analyzed (Fig 3C), a random distribution was observed in most of the evaluations, except the 5^th^, 15^th^ and 18^th^ evaluations, with an aggregate distribution, and the 17^th^ evaluation, with a uniform distribution.

### Optimum sample size and sampling scheme

**Fig 4:**
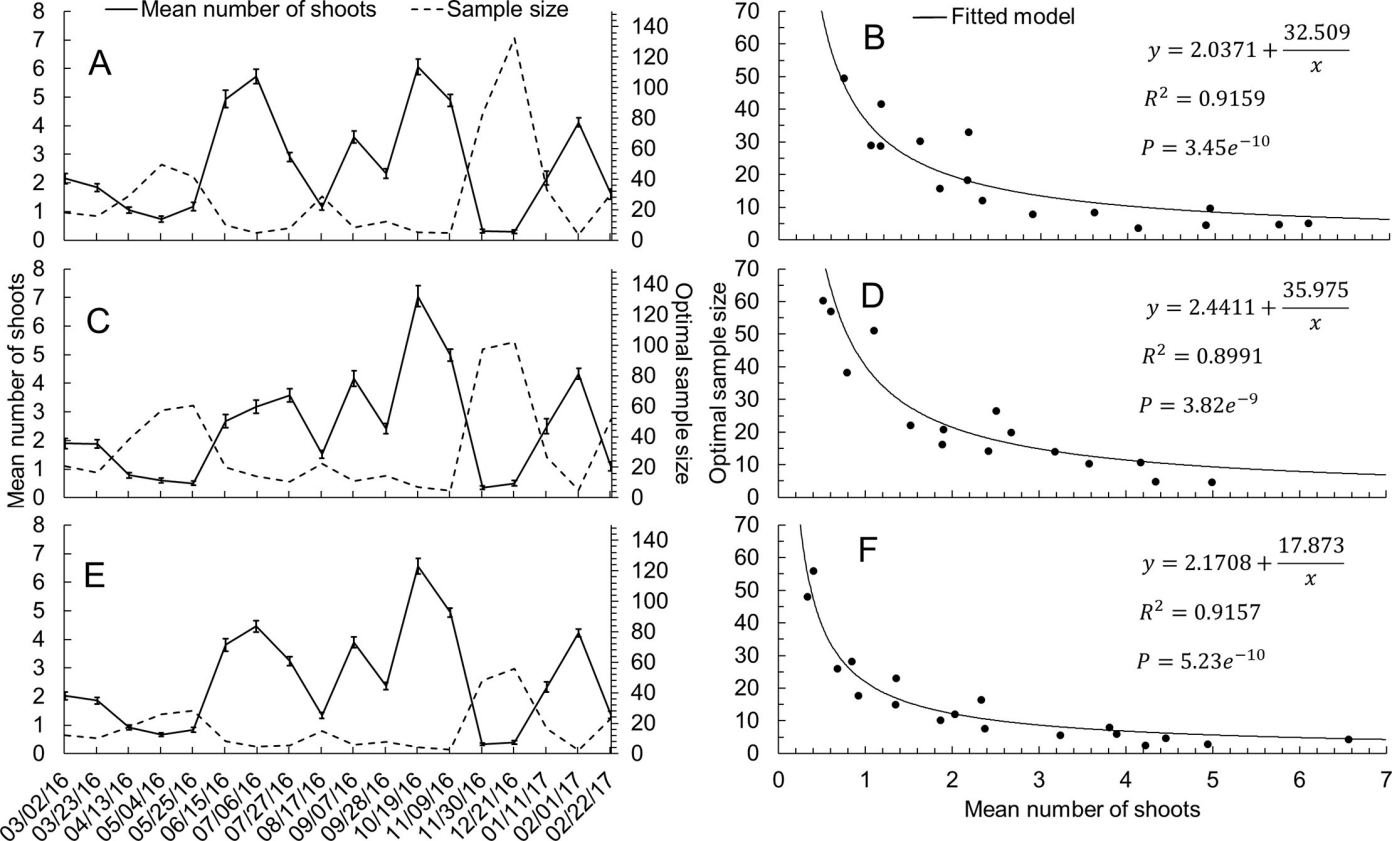
Minimum number of trees to be sampled using the mean number of new shoots. Optimal sample size (number of trees) to reach a relative sampling error of 25% [$n={\left(s/E_{r}\bar{x}\right)}^{2}$] [3] and mean number of shoots (±SEM) on each sample date for each sample size, considering the average number of shoots inside the 0.25 m^2^ frame [west side (A and B), east side (C and D), average data from both sides (E and F)].

The seasonal variation of the occurrence of new shoots indicated that the number of trees to be sampled to reach a given relative sampling error (*E*_*r*_) varied over the assessment dates and between the east and west sides of the canopy (Fig 4A to 4F). Although reproductive shoots were not considered for phenological classification, in the flowering season the average number of reproductive shoots counted within a square frame was 6.28 per tree.

The optimal sample size was negatively correlated with the mean number of new shoots, regardless of the side of the canopy. On the west side, considering an *E*_*r*_ of 5%, the number of trees ranged between 93 and 3,311 (mean = 720). However, considering a more flexible but still acceptable level of 15% or 25% [11], the sample size ranged from 10 to 368 (mean = 80) and 4 to 132 (mean = 29) trees, respectively (Fig 4A and 4B). On the east side, the optimum sample size for a *Er* of 5%, 15% or 25% was slightly higher than for the west side, varying between 117 and 2,552 (mean = 804), 13 and 284 (mean = 89), and 5 and 102 (mean = 32; Fig 4C and 4D), respectively, possibly due to a less uniform distribution of flush shoots on the west side. Considering the averages of both sides, the optimum number of trees to be sampled was even smaller, with 61 to 1,398 (mean = 410), 7 to 155 (mean = 46), and 2 to 56 (mean = 16; Fig 4E and 4F) to achieve *E*_*r*_ of 5%, 15%, and 25%, respectively.

**Fig 5:**
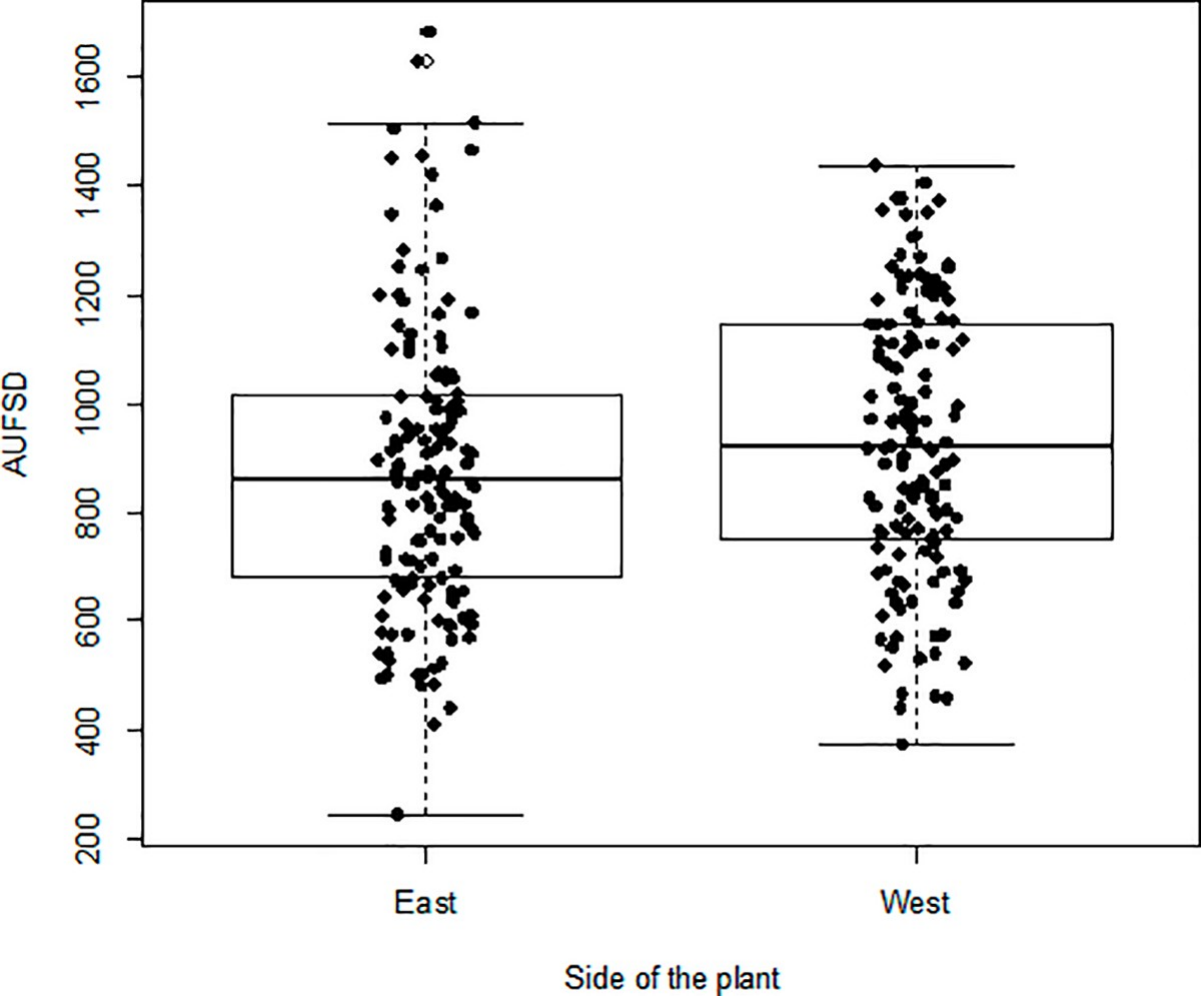
The AUFSD calculated for each sampled tree. Boxplots the AUFSD from both sides of the canopy of ‘Valencia Late’ sweet orange trees grafted onto ‘Swingle’ citrumelo rootstock.

The estimated AUFSD considered only the trees that remained healthy in appearance until the end of the experiment (141 out of 160 trees). This allowed simplifying the analysis, since it combines the intensity and frequency values of flush shoots in a single value. Initially, the data from both sides of the tree followed a normal distribution (Shapiro-Wilk = 0.98; P = 0.11) and were homoscedastic (*F* = 0.99; *P* = 0.94). There was significant difference for the AUFSD between the west side, with avg. = 938.25 NS-days and *σ* = 255.87, and the east side, with avg. = 869.11 NS-days and *σ* = 258.00 (paired *t-*test = 2.42; *P* = 0.0169; Fig 5).

**Fig 6:**
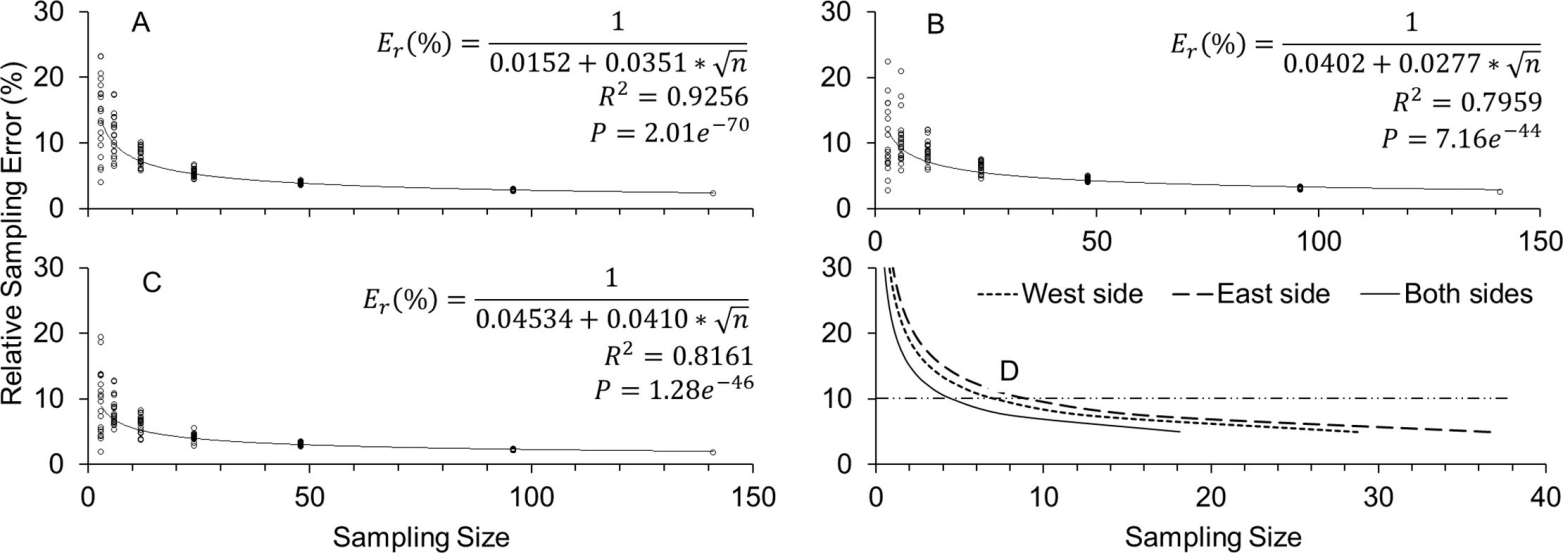
Minimum number of trees to be sampled using area under the flush shoot dynamics curve (AUFSD). Relative sampling error (*E*_*r*_) of the area under the flush shoot dynamics curve as a function of sample size. Each empty point represents one set of trees randomly selected (from a total of 20 sets) with n = 3, 6, 12, 24, 48 and 96 trees (A: west side; B: east side; C: average data from both sides). D: Optimum sample size (number of trees) needed to achieve a given *E*_*r*_ $\left({n=[\sigma /\left(E_{r}*\bar{X}\right)]}^{2}\right)$ [3], by considering the area under the curve of new shoot number for mature ‘Valencia Late’ sweet orange trees grafted onto ‘Swingle’ citrumelo rootstock. A more flexible *E*_*r*_ of 10% is indicated by the horizontal line.

Validation of the data, which included 20 sets of randomly selected trees with sample sizes of 3 to 96, totaling 120 data points, also showed a slightly lower optimum tree sample size for the west side compared with the east side (Fig 6A and 6B). Similarly, the optimum sample size for the AUFSD data from both sides was lower than those for either the west or east side (Fig 6C). The *E*_*r*_ values on the west, east, and both sides were 14% (4.02–23.19), 13% (2.73–35.79) and 10% (1.87–19.38) when 3 trees were sampled, and 8% (5.74–10.09), 9% (5.90–12.06) and 6% (3.66–8.20) when 12 trees were sampled (Fig 6A to 6C). This estimation suggested that by considering only the west side, the number of trees to be sampled would be lower than for the east side (-22%; Fig 6D). However, if both sides of the tree are sampled and values of AUFSD are averaged, the optimum sample sizes would be 37% and 51% lower than those for the west and east side, respectively (Fig 6D). To reach *E*_*r*_ of 5%, it would be necessary to sample 29 trees on the west and 37 on the east side, or 18 trees with the average data from both sides. A more flexible *E*_*r*_ (e.g., 10% as indicated by the horizontal line in Fig 6D), would give optimum sample sizes of 7 and 9 trees for the west and east sides, respectively, or 5 trees for the average of both sides.

Considering the optimal sample size of 16 trees evaluated on both sides of the canopy for *E*_*r*_ = 25%, and selected in the block according with eight different transects (Table 2), the values of mean number of shoots in the frame for all but transects 1 to 4 (very concentrated sampled trees in different sectors of the block, in contrast to more distributed sampled trees in transects 5 to 8) were very close to the general average obtained from all 160 trees sampled within the area. The average absolute difference between all 160 trees and average of transects 1 to 4 or 5 to 8 were 10.2% (max.: 21.11% and min.: 1.98%) and 2.7% (max.: 5.46% and min.: 1.05%), respectively (Table 2).

**Table 2:** Mean number of shoots in the frame considering all trees and the optimal sample size of 16 trees by means of eight different transects, evaluated on both sides of the canopy within the area for *E*_*r*_ of 25%.

Date of assessment	All trees	Transects							
		I	II	III	IV	V	VI	VII	VIII
03/02/2016	2.039	3.093	1.593	1.500	1.843	2.187	2.233	1.250	1.343
03/23/2016	1.929	2.312	1.593	2.437	1.437	2.093	1.766	1.437	1.781
04/13/2016	0.904	1.125	0.437	1.187	0.718	1.156	0.866	1.031	0.562
05/04/2016	0.656	0.156	0.718	0.406	1.093	0.437	0.733	0.500	0.406
05/25/2016	0.812	0.906	0.375	0.750	1.125	0.218	0.933	0.718	0.843
06/15/2016	3.836	5.843	2.031	4.312	3.343	4.375	2.700	4.031	3.937
07/05/2016	4.556	5.437	4.000	4.781	4.062	4.687	2.866	5.093	5.125
07/27/2016	3.305	2.781	3.562	3.500	3.625	4.031	3.766	3.406	3.531
08/17/2016	1.397	1.062	1.343	1.406	2.000	1.156	2.333	1.593	1.218
09/07/2016	3.833	5.781	2.812	4.875	3.156	4.156	3.933	4.812	3.562
09/28/2016	2.329	3.531	1.437	2.750	2.343	2.531	2.000	2.687	2.343
10/19/2016	6.673	10.625	6.625	6.531	5.343	6.062	6.800	6.281	5.531
11/09/2016	4.929	2.437	5.781	5.312	5.906	4.875	5.866	4.593	4.500
11/30/2016	0.329	0.187	0.125	0.593	0.312	0.468	0.200	0.375	0.625
12/21/2016	0.404	0.218	0.406	0.281	0.687	0.218	0.700	0.656	0.312
01/11/2017	2.067	3.531	2.281	1.750	1.562	2.156	2.200	2.718	1.875
02/01/2017	4.280	4.406	4.125	4.031	4.531	4.656	4.333	4.156	4.218
02/22/2017	1.464	1.968	0.312	1.312	1.750	1.406	1.033	1.218	1.531
General Average	2.541	3.078	2.197	2.651	2.491	2.604	2.514	2.586	2.402

### Distribution of the shoots in the canopy

**Fig 7:**
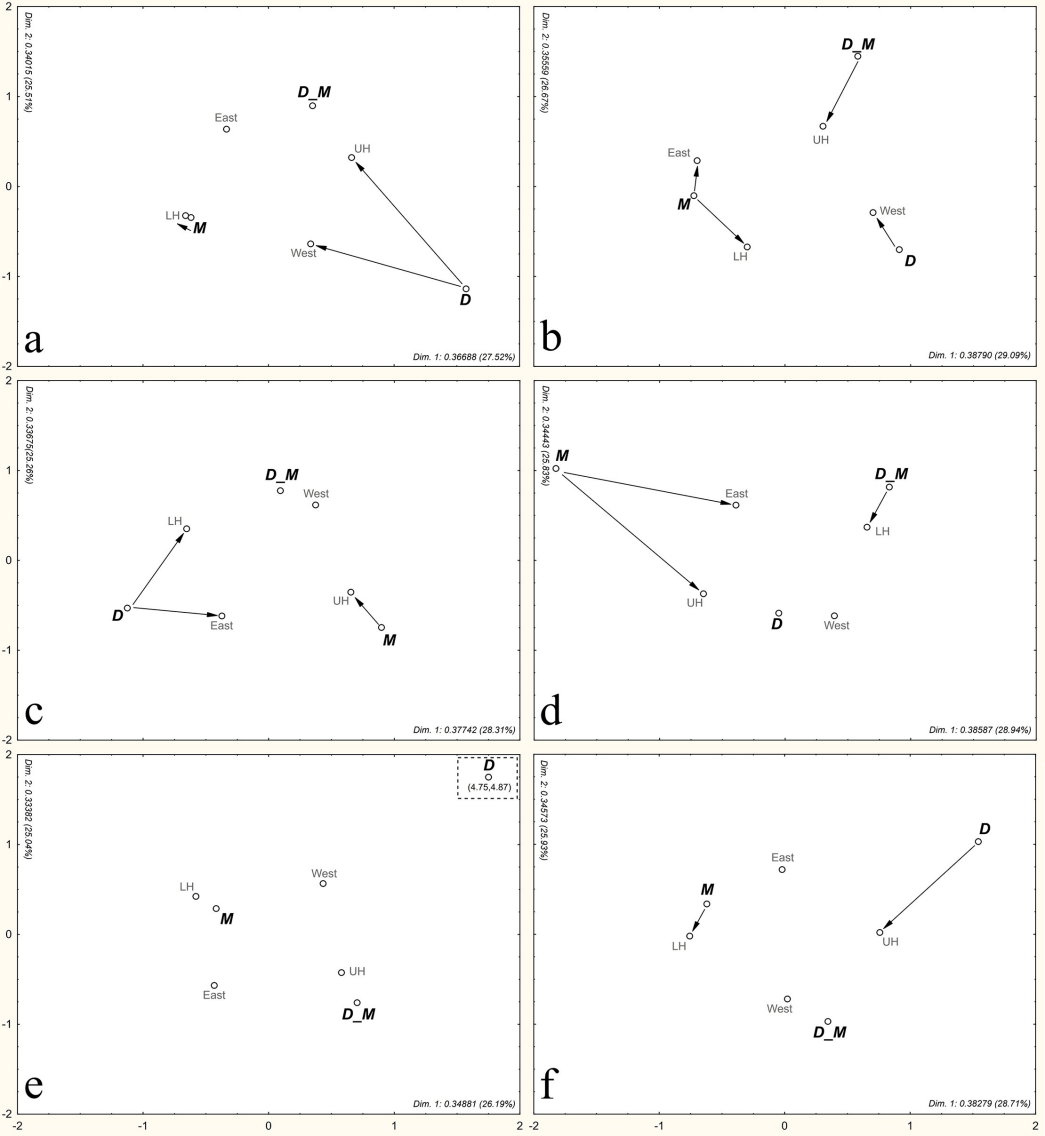
Distribution of the shoots in the canopy. Seasonal variation of the multiple correspondence analysis of relationships between the categories of side of the tree (West or East side), each strata (UH: upper half; LH: lower half), and the grouped developmental phase of new shoots (D: development, V2 and V3; D_M: development to maturation, V4 and V5; M: completely mature shoots, V6 and V7), in each assessment period (a: 1^st^, 2^nd^, and 3^rd^; b: 4^th^, 5^th^, and 6^th^; c: 7^th^, 8^th^, and 9^th^; d: 10^th^, 11^th^, and 12^th^; e: 13^th^, 14^th^, and 15^th^; f: 16^th^, 17^th^, and 18^th^). Arrows indicate significant (p < 0.05) correspondence between categories.

The multiple correspondence analysis showed significant correspondence between the categories on each of the evaluation dates (*X*^*2*^: 15377 to 15600, df = 36, *P* < 0.0001). Overall, MCA of the combined data every three assessment dates allowed observing that the phenological stage of the predominant shoot was different between the sides of the canopy as well as for each strata (upper or lower half).The first two dimensions of the MCA represented more than 50% of the inertia (Fig 7A–7F and S2 Fig).

A preliminary analysis comparing the left quadrant with the right and the upper part with the lower part of the canopy, showed more association between the growth stage of the shoots and the upper or lower part of the canopy than with the left or right quadrants. So, we decided to combine the samples from the upper half, on the one hand, and the samples from the lower part, on the other, for analysis.

Generally, the west side and the upper half of the trees were more frequently associated with shoots in fast growth (v1 to v3), an indication of recent start of the flushing event, and with shoots in the process of maturation (v4 to v5), whereas the east side and the lower half of the trees were most frequently associated with maturing or fully mature shoots (v6 to v7), regardless of the date of observation (Fig 7A–7F).

In the late summer mature shoots were more associated with lower half, whereas developing shoots with west side and upper half of the canopy (Fig 7A). In early autumn (Fig 7B), the young shoots were more likely to be present in the west side, the *D_M* shoots in the upper half of the tree, and the mature shoots in the east and lower half of the canopy. In the winter, developing shoots were more commonly found in the east side and lower half, whereas mature shoots in the upper half of the canopy (Fig 7C). In the early spring (Fig 7D), *D_M* shoots were more associated with lower half, whereas mature shoots with east side and upper half of the trees. In the late spring (Fig 7E), none of the *D_M* or mature shoots was significantly associated with any sector of the tree. Finally, in the last sampling period the behavior was similar to the beginning of the experiment, with shoots in development associated with the upper half of the trees and the mature shoots with the lower half.

### Overall shoot maturity index

**Fig 8:**
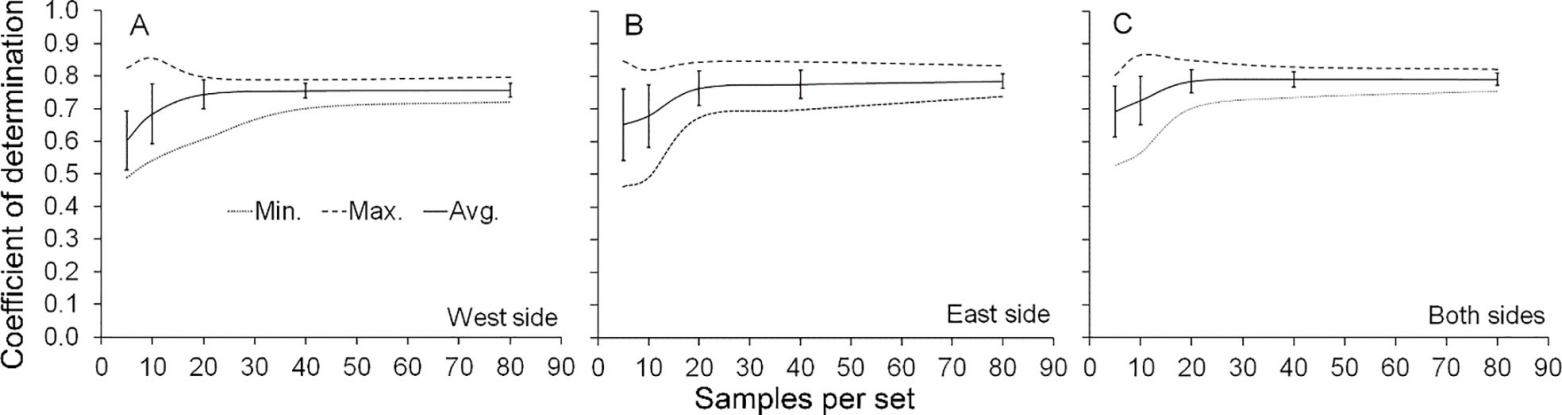
Minimum number of tree samples using the shoot maturity index. Minimum (min.), maximum (max.), averages (avg.) and standard deviation (±SD) values of the coefficient of determination (*R*^*2*^) from 20 sets of data with randomly selected sample sizes (n) of 5, 10, 20, 40 and 80 trees. *R*^*2*^ values from the entire experiment (160 trees) were 0.7667, 0.7959 and 0.7963 for west side (A), east side (B), and average of both sides (C).

The shoot maturity index (SMI) was correlated negatively with the mean number of new shoots within the square frame, regardless of the side of the tree considered (S3 Fig). The lowest coefficient of determination was obtained when 5 trees on the east side were sampled, while the maximum was obtained with the average data from both sides of 10 trees (Fig 8A to 8C). Considering the average of the 20 sets of randomly generated data, the lowest coefficient of determination (*R*^*2*^ = 0.7535) was obtained with 5 trees in the west side (Fig 8A), as shown (S3A Fig), while the largest (*R*^*2*^ = 0.8108) was obtained with the average number of shoots counted on both sides of the canopy (Fig 8C) and the sum of the SMI for all 8 sampling positions of the canopy (S3I Fig).

On average, new shoot counts decreased at a rate of 0.32 shoot per one-unit increment of the SMI when considering only one side of the canopy, or 0.16 shoot when considering the mean of both sides (S3 Fig). Regarding the number of trees to be sampled to determine the SMI, Fig 4 shows that regardless of the sampled side (even the whole tree), there was no significant improvement of the relationship between the shoot maturity index and the mean number of new shoots counted inside the projection of the 0.25 m^2^ square frame (increase in the *R*^*2*^) beyond 20 trees per sample (Fig 8).

## Discussion

Evaluating the shoot growth of citrus trees is important in studies on crop physiology and production, as well as for germplasm evaluation. In addition, this information could improve the criteria for pest management procedures, for instance by the means of determining periods of higher host favorability, since flushes are a main entry way for several pathogens of citrus. Therefore, it is necessary to formulate methods for growers and researchers to adequately evaluate the presence, distribution and temporal dynamics of new flush shoots. In this work, both quantitative and qualitative methods were used to examine statistical techniques to estimate the minimum number and location of trees to be sampled and to establish the temporal and spatial distribution pattern of the flush shoots in ‘Valencia Late’ orange trees.

This work was carried out in the north of SPS, the state with the largest citrus growing area in Brazil, using ‘Valencia Late’ sweet orange, which accounts for 27% of production from commercial orchards [36]. Trees were grafted onto ‘Swingle’ citrumelo rootstock, used in 50% of SPS citrus nurseries production in 2016 [37]. These scion and rootstock varieties are also wide cultivated in other main citrus producing regions in the world [38,39]. The crop management practices, soil and climate conditions of the experimental area were typical for citrus cultivation in SPS [40]. Therefore, we can assume that representative scion/rootstock combination and citrus cultivation conditions under a tropical environment were studied in this work. Different citrus species and varieties could show distinct flushing behavior, such as lemons [*C*. *limon* (L.) Burm. f] and ‘Persian’ lime [*C x latifolia* (Yu. Tanaka) Tanaka] that develop new shoots year round while flushes seem to be more defined in sweet oranges trees [2], and thus specific adjustments of the developed methods may be necessary. However it is unlikely that a smaller number of tree samples will be needed in such cases, and although more trees would provide more accurate results, the optimal number of trees to sample would not substantially vary from our results, since there is no practical means to scout such a large number of trees in the field.

We found a higher number of shoots on the west side of the canopy (Fig 5), which could be related to higher incidence of sunlight and higher sap flow, as happens in other plants [41–43]. This could also be associated with the temporal and spatial variations in phloem sap flow in response to sunlight exposure, as seen for other woody species like *Cryptomeria japonica* [44] and *Pinus taeda* [45]. Unlike our results, Ramos et al. [46] did not find azimuthal variation in the number of new shoots in ‘Hamlin’ sweet orange trees grafted on ‘Swingle’ citrumelo rootstock, probably due to methodological differences (two branches per tree vs. square frame on both sides) or age of the trees (three vs. five years old). Azimuthal variations in flush shoots and its relation to population densities of ACP nymphs were reported in Texas [47].

Counting new shoots on two sides of the canopy can be more effective for flush monitoring aiming at pest control, since a single assessment would give the status of the heterogeneous population of new shoots in the orchard. Despite the fact that in SPS there are seasons with a greater population of new shoots (e.g., late winter–early spring, Fig 4), we recommend to monitor flushes throughout the year, because during other seasons with a lower flush shoot population, there may still be suitable flushes for feeding, oviposition, and development of insect vectors. Moreover, the evaluation of only vegetative shoots facilitated monitoring of phenological stages and provided reliable sampling. Although during the flowering season about 50% of shoots were reproductive (mixed-type), having included them in the analyzes would not have made much difference, since the average number of shoots would have been greater than 6 and, as seen in Fig 4, there would have been no significant reduction in sample size.

According to the Taylor's power law coefficients, the new shoots were mostly randomly distributed in time and in space, especially for the west side or for the averaged data from both sides. On the east side, the shoots were aggregated on four assessment dates and uniformly distributed on two. That characteristic happened during periods with low shoot population and lower temperatures. The predominantly random distribution of the new shoots in trees within the block implies that any tree can be considered for sampling, since the probability of finding a new shoot in a tree in any location within the block is the same, and the presence of a given new shoot does not influence the presence of another one [9]. However, there are some aspects of the sampling scheme that must be considered, such as establishing a minimum distance from the edges of the block, avoiding flooded areas, selecting trees that represent an approximation of the vigor and architectural status of most plants, and assuring physical and chemical soil homogeneity and uniform topography, among other factors. In addition, as showed by the testing different sampling schemes, the more distributed the selected trees within the block, the closer the sample mean was to the population mean (S1 Appendix and Table 2). When some of these conditions are not satisfied, the need to subdivide the surveyed blocks should be considered.

In our study, with the use of a square frame of 0.25 m^2^ the number of trees to be sampled was lower than that obtained by Hall and Albrigo [12] using a 3.47 dm^3^ frame, 40 vs 16 trees. The reason could be the higher probability of finding more shoots within the larger area provided by our square frame [18]. Interestingly, the area under the curve of the flush shoot dynamics (AUFSD), which was estimated over the entire evaluation period to combine in a single value the intensity and temporal dynamic of flush shoots, yielded even lower sample size. From a population of 141 trees, the AUFSD showed that it would be necessary to sample 7 trees on the west side, 9 trees on the east side or 5 trees each on both sides to reach *E*_*r*_ of 10%. These numbers are smaller compared to those obtained with the counts of mean number of new shoots, which required 29, 32 and 16 for the west, east and both sides, respectively, to reach *E*_*r*_ of 25%. Thus, the AUFSD could be useful for research purposes, since it is desirable to assess a lower number of trees with a small *E*_*r*_, which provides higher data reliability. This is particularly important when the aim is standardized long-term evaluation of citrus germplasm, for instance, in field studies that investigate the host-vector relationship within citrus trees and related genera [48].

The multiple correspondence analysis (MCA) indicated an overall higher frequency of younger shoots on the west side and in the upper sampling positions of the canopy, and older shoots on the opposite side. Feeding and reproductive behavior of some citrus pests are strongly associated with the developmental stage of the new shoots [7, 49–51]. As a result, the upper west could be the most appropriate side to install yellow sticky cards for ACP monitoring.

The high correlation between the qualitative method using the shoot maturity index (SMI), which was based on the type of new flush predominant in the tree, and the mean number of new shoots makes the SMI a reliable measure to estimate the overall level of vulnerability of the grove to vectors. The accuracy of the correlation remained high and stable above sample sizes of 20 trees (Fig 8A to 8C), giving an extra advantage to the visual assessment. Although no cost/efficiency relationship analysis between methods was carried out, monitoring with the SMI was clearly faster than shoot counting, allowing assessing a higher number of trees at the same time. Data collection by this method is straightforward and minimizes time, cost, and degree of skill required for monitoring flush shoots with a lower risk of compromising accuracy and precision.

It is important to note that these results are more applicable to smaller and homogeneous areas, since we used a uniform orchard with a total number of 833 trees (ca. 1 ha). However, regarding commercial groves in SPS, for instance, which usually contain around 15,000 plants per block, shoot monitoring is clearly a laborious task in field circumstances of larger areas because it will be necessary to sample a significant number of trees regardless the monitoring method used. In the near future, automated methods that require more advanced technologies, such as the images by drones, could provide fast, precise and inexpensive area-wide monitoring of shoot flushing in commercial citrus orchards [52].

In conclusion, sampling flush shoots in orange trees can be influenced by the objective of the research and by the statistical method employed. Shoot monitoring should be performed throughout the seasons. The AUFSD metric enabled using a smaller sample, of at least five trees on both sides, with lower relative variation of data, and therefore is suggested for continuing studies. The SMI has the potential to monitor flush shoots in the field more efficiently, given the feasibility of visual assessments, providing a correlation high and stable above sample sizes of 20 trees. Furthermore, the young shoots are located mainly in the upper part of the canopy as well as the side of the canopy more exposed to sunlight, randomly occurring throughout the year, as revealed by the spatial and temporal analysis, respectively.

## Supporting information

S1 Appendix Different sampling schemes tested. (XLSX)

S2 Appendix Raw data from the quantitative methods. (XLSX)

S3 Appendix Raw data from qualitative methods. (XLSX)

S1 Table Spatial distribution of new shoots. Parameter estimates (±SE) of Taylor’s power law from the linear regression of the log-transformed data of variance against the mean for determination of the spatial distribution, and Student’s *t*-test for each coefficient on each assessment date and p-value of the H_0_.
(PDF)

S1 Fig Distribution of the sampling positions in the canopy during the evaluation period. The canopy was divided into eight sampling positions: four on each side, two in the upper (SP1 and SP2) and two in the lower half (SP3 and SP4) of west face of the plant in the planting line; two in the upper (SP5 and SP6) and two in the lower half (SP7 and SP8) for east face.
(PDF)

S2 Fig Distribution of the shoots in the canopy during the evaluation period. Bi-dimensional representation of the MCA (first two dimensions) on the association between different levels of the categorical variables (Side of the plant: S1 [west] or S2 [east]; sampling position of the canopy: upper or lower half (UH, LH): developing (D), developing-maturing (D_M), or completely mature (M)), for each assessment date (a, 02/03/16; b, 23/03/16; c, 13/04/16; d, 04/05/16; e, 25/05/16; f, 15/06/16; g, 05/07/16; h, 27/07/16; i, 17/08/16; j, 07/09/16; k, 28/09/16; l: 19/10/16; m: 09/11/16; n: 30/11/16: o: 21/12/16; p:11/01/17; q: 01/02/17; r: 21/02/17).
(PDF)

S3 Fig Overall shoot maturity index. Relationship between the mean number of new shoots inside the projection of the 0.25 m^2^ square frame in the outer center of the canopy and the maturity index estimated by the predominant flush stage in each sampling position and each side of the canopy (see Materials and Methods for more details). Each point in the plots represents one assessment date and corresponds to the average of 20 sets of randomly selected trees with n = 5 (A, B, C), 10 (D, E, F), 20 (G, H, I), 40 (J, K, L), 80 (M, N, O) trees. The estimated relationship with 160 trees (P, Q, R) is also included.
(PDF)
