# Supplementary material for: Optimization of sampling and monitoring of vegetative flushing in citrus orchards
Source: PLoS One. 2020 May 20;15(5):e0233014. doi: 10.1371/journal.pone.0233014 (PMC7239491; doi:10.1371/journal.pone.0233014)
Supplement: S1 Table — Parameter estimates (±SE) of Taylor’s power law from the linear regression of the log-transformed data of variance against the mean for determination of the spatial distribution, and Student’s t-test for each coefficient on each assessment date and p-value of the H0. (PDF) [file pone.0233014.s004.pdf]

S1 Table. Parameter estimates (±SE) of Taylor's power law from the linear regression of the log-transformed data of variance against the mean for determination of the spatial distribution, and Student's *t*-test for each coefficient on each assessment date and p-value of the H_0.

Assessment	Parameter	West Side			East Side			Both Sides		
		Value	H_0: $\beta \neq 0$[a]	H_0: $\beta \neq 1$[b]	Value	H_0: $\beta \neq 0$[a]	H_0: $\beta \neq 1$[b]	Value	H_0: $\beta \neq 0$[a]	H_0: $\beta \neq 1$[b]
1	β_0	0.81(0.16)	**$4.97e^{-5}$**	0,1786	0.58(0.20)	**0,0091**	**0,0373**	0.43(0.18)	**0,0262**	**0,0015**
	β_1	0.94(0.19)	**$6.78e^{-5}$**	0,3702	1.39(0.28)	**$7.75e^{-5}$**	0,1495	0.84(0.24)	**0,0021**	0,3145
2	β_0	0.80(0.14)	**$9.6e^{-6}$**	0,1119	0.68(0.02)	**$2.69e^{-53}$**	**$1.01e^{-208}$**	0.15(0.27)	0,5889	**0,0018**
	β_1	0.51(0.20)	**0.0178**	**0,0134**	0.96(0.03)	**$1.75e^{-223}$**	0,1467	0.68(0.39)	0,0978	0,2784
3	β_0	0.60(0.09)	**$4.28e^{-6}$**	**$1.63x10^{-5}$**	0.62(0.04)	**$8.39e^{-14}$**	**$2.86e^{-35}$**	-0.15(0.10)	0,1566	**$1.29e^{-29}$**
	β_1	0.81(0.19)	**$4.46e^{-4}$**	0,2248	0.77(0.06)	**$3.86e^{-11}$**	**$2.73e^{-5}$**	0.68(0.17)	**$6.19e^{-4}$**	0,0516
4	β_0	0.73(0.12)	**$3.63e^{-6}$**	**0,0171**	0.82(0.04)	**$1.78e^{-15}$**	**$3.99e^{-8}$**	-0.04(0.14)	0,8093	**$1.23e^{-13}$**
	β_1	1.30(0.10)	**$1.03e^{-10}$**	**0,0040**	1.25(0.07)	**$1.52e^{-13}$**	**0,0003**	1.22(0.14)	**$4.34e^{-8}$**	0,1116
5	β_0	0.74(0.10)	**$7.53e^{-7}$**	**0,0110**	1.24(0.06)	**$9.55e^{-15}$**	**$3.04e^{-5}$**	0.26(0.14)	0,0808	**$1.99e^{-7}$**
	β_1	1.57(0.14)	**$2.48e^{-9}$**	**$8.98x10^{-5}$**	1.13(0.09)	**$8.63e^{-11}$**	0,1352	1.41(0.19)	**$4.46e^{-7}$**	**0,0337**
6	β_0	0.69(0.35)	0.0646	0,2635	2.42(0.10)	**$2.00e^{-15}$**	**$4.69e^{-48}$**	0.25(0.39)	0,5323	0,0616
	β_1	1.09(0.23)	**$1.15e^{-4}$**	0,3725	-0.23(0.10)	**0,0249**	**$5.57e^{-39}$**	1.01(0.30)	**0,0030**	0,3988
7	β_0	0.77(0.93)	0.4210	0,3862	1.17(0.20)	**$8.93e^{-6}$**	0,2757	0.60(0.96)	0,5434	0,3637
	β_1	0.81(0.54)	0.1474	0,3739	0.82(0.16)	**$7.54e^{-5}$**	0,2050	0.64(0.65)	0,3335	0,3398
8	β_0	1.09(0.58)	0.0771	0,3948	0.86(0.09)	**$3.34e^{-9}$**	0,0730	-0.18(0.56)	0,7538	**0,0432**
	β_1	0.26(0.55)	0.6493	0,1527	1.12(0.09)	**$1.09e^{-10}$**	0,1529	1.05(0.48)	**0,0419**	0,3972
9	β_0	0.75(0.06)	**$1.75e^{-11}$**	**$1.40x10^{-6}$**	1.03(0.11)	**$1.32e^{-8}$**	0,3908	0.12(0.11)	0,3067	**$1.96e^{-16}$**
	β_1	0.67(0.10)	**$2.34e^{-6}$**	**0,0014**	1.12(0.10)	**$4.60e^{-10}$**	0,1809	0.95(0.20)	**$1.73e^{-4}$**	0,3817
10	β_0	0.19(0.39)	0.6253	**0,0412**	0.40(0.09)	**0,0001**	**$2.14e^{-13}$**	0.34(0.63)	0,6026	0,2258
	β_1	1.24(0.30)	**$6.04e^{-4}$**	0,2903	1.41(0.07)	**$7.46e^{-14}$**	**$1.84e^{-8}$**	0.83(0.47)	0,0921	0,3719
11	β_0	0.52(0.27)	0.0676	0,0732	0.72(0.09)	**$5.40e^{-8}$**	**0,0008**	0.01(0.36)	0,9784	**0,0088**
	β_1	0.91(0.31)	**0.0083**	0,3797	1.04(0.12)	**$3.92e^{-8}$**	0,3779	0.74(0.41)	0,0846	0,3238
12	β_0	-1.69(0.61)	**0.0126**	**$2.37x10^{-5}$**	-0.11(0.59)	0,8531	0,0645	-0.84(0.84)	0,3311	**0,0353**
	β_1	2.12(0.35)	**$7.60e^{-6}$**	**0,0019**	1.46(0.31)	**0,0001**	0,1309	1.51(0.45)	**0,0033**	0,2087
13	β_0	0.05(0.65)	0.9399	0,1332	1.20(0.50)	**0,0270**	0,3700	0.01(0.84)	0,9986	0,1965
	β_1	0.98(0.41)	**0.0281**	0,3982	0.28(0.32)	0,3838	**0,0270**	0.61(0.53)	0,2645	0,3039
14	β_0	0.40(0.24)	0.1246	**0,0157**	0.78(0.18)	**0,0009**	0,1674	-0.26(0.26)	0,3368	**$2x84e^{-6}$**
	β_1	1.00(0.19)	**$1.20e^{-4}$**	0,3989	1.38(0.17)	**$3.75e^{-6}$**	**0,0266**	1.05(0.20)	**$7.22e^{-5}$**	0,3898
15	β_0	1.25(0.18)	**$9.39e^{-6}$**	0,1517	0.86(0.11)	**$1.67e^{-6}$**	0,1528	0.27(0.16)	0,0984	**$3.67e^{-6}$**
	β_1	1.61(0.14)	**$2.69e^{-8}$**	**$2.28x10^{-5}$**	1.37(0.08)	**$1.82e^{-10}$**	**$5.85e^{-6}$**	1.30(0.10)	**$1.20e^{-10}$**	**0,0051**
16	β_0	0.89(0.17)	**$5.56e^{-5}$**	0,3226	0.85(0.21)	**0,0006**	0,2963	0.13(0.18)	0,4793	**$2x87e^{-6}$**
	β_1	1.35(0.18)	**$5.15e^{-7}$**	0,0610	1.37(0.22)	**$4.70e^{-6}$**	0,0900	1.38(0.20)	**$1.43e^{-6}$**	0,0617
17	β_0	0.81(1.44)	0.5795	0,3954	1.32(1.07)	0,2343	0,3822	0.92(1.63)	0,5815	0,3984
	β_1	0.25(1.02)	0.8097	0,3037	0.16(0.73)	0,8392	0,2026	-0.08(1.14)	0,9446	0,2529
18	β_0	0.75(0.12)	**$5.77e^{-6}$**	**0,0378**	0.91(0.16)	**$4.52e^{-5}$**	0,3265	0.38(0.12)	**0,0044**	**$1.57e^{-7}$**
	β_1	1.33(0.17)	**$3.18e^{-7}$**	0,0633	1.43(0.26)	**$6.74e^{-5}$**	0,0998	1.37(0.16)	**$6.26e^{-8}$**	**0,0253**

[a] Student's *t* test; [b] statistical significance was determined as 1 $\notin$ ±95% CI; significant values are in bold.
