# Supplementary material for: Optimization of sampling and monitoring of vegetative flushing in citrus orchards
Source: PLoS One. 2020 May 20;15(5):e0233014. doi: 10.1371/journal.pone.0233014 (PMC7239491; doi:10.1371/journal.pone.0233014)
Supplement: S1 Fig — The canopy was divided into eight sampling positions: four on each side, two in the upper (SP1 and SP2) and two in the lower half (SP3 and SP4) of west face of the plant in the planting line; two in the upper (SP5 and SP6) and two in the lower half (SP7 and SP8) for east face. (PDF) [file pone.0233014.s005.pdf]

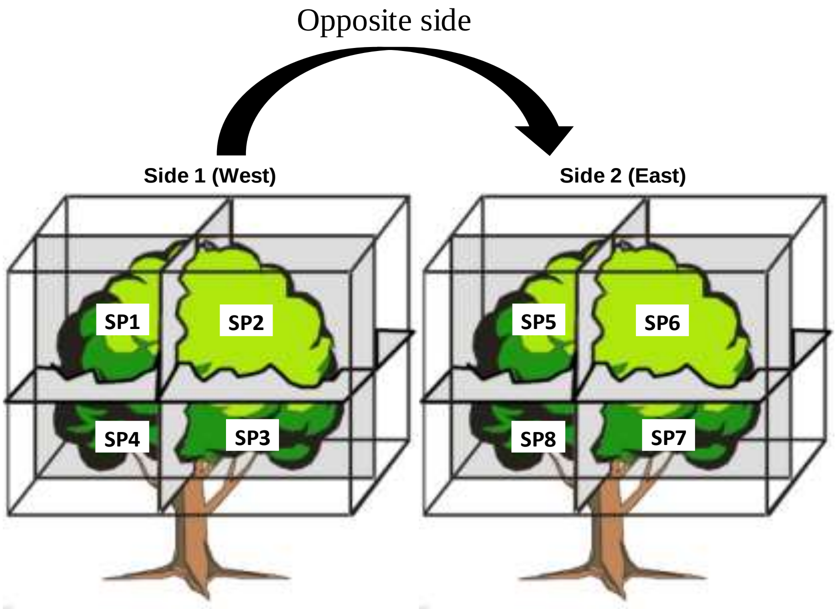

S1 Fig. Distribution of the sampling positions in the canopy during the evaluation period. The canopy was divided into eight sampling positions: four on each side, two in the upper (SP1 and SP2) and two in the lower half (SP3 and SP4) of west face of the plant in the planting line; two in the upper (SP5 and SP6) and two in the lower half (SP7 and SP8) for east face.
