# Supplementary material for: Optimization of sampling and monitoring of vegetative flushing in citrus orchards
Source: PLoS One. 2020 May 20;15(5):e0233014. doi: 10.1371/journal.pone.0233014 (PMC7239491; doi:10.1371/journal.pone.0233014)
Supplement: S2 Fig — Bi-dimensional representation of the MCA (first two dimensions) on the association between different levels of the categorical variables (Side of the plant: S1 [west] or S2 [east]; sampling position of the canopy: upper or lower half (UH, LH): developing (D), developing-maturing (D_M), or completely mature (M)), for each assessment date (a, 02/03/16; b, 23/03/16; c, 13/04/16; d, 04/05/16; e, 25/05/16; f, 15/06/16; g, 05/07/16; h, 27/07/16; i, 17/08/16; j, 07/09/16; k, 28/09/16; l: 19/10/16; m: 09/11/16; n: 30/11/16: o: 21/12/16; p:11/01/17; q: 01/02/17; r: 21/02/17). (PDF) [file pone.0233014.s006.pdf]

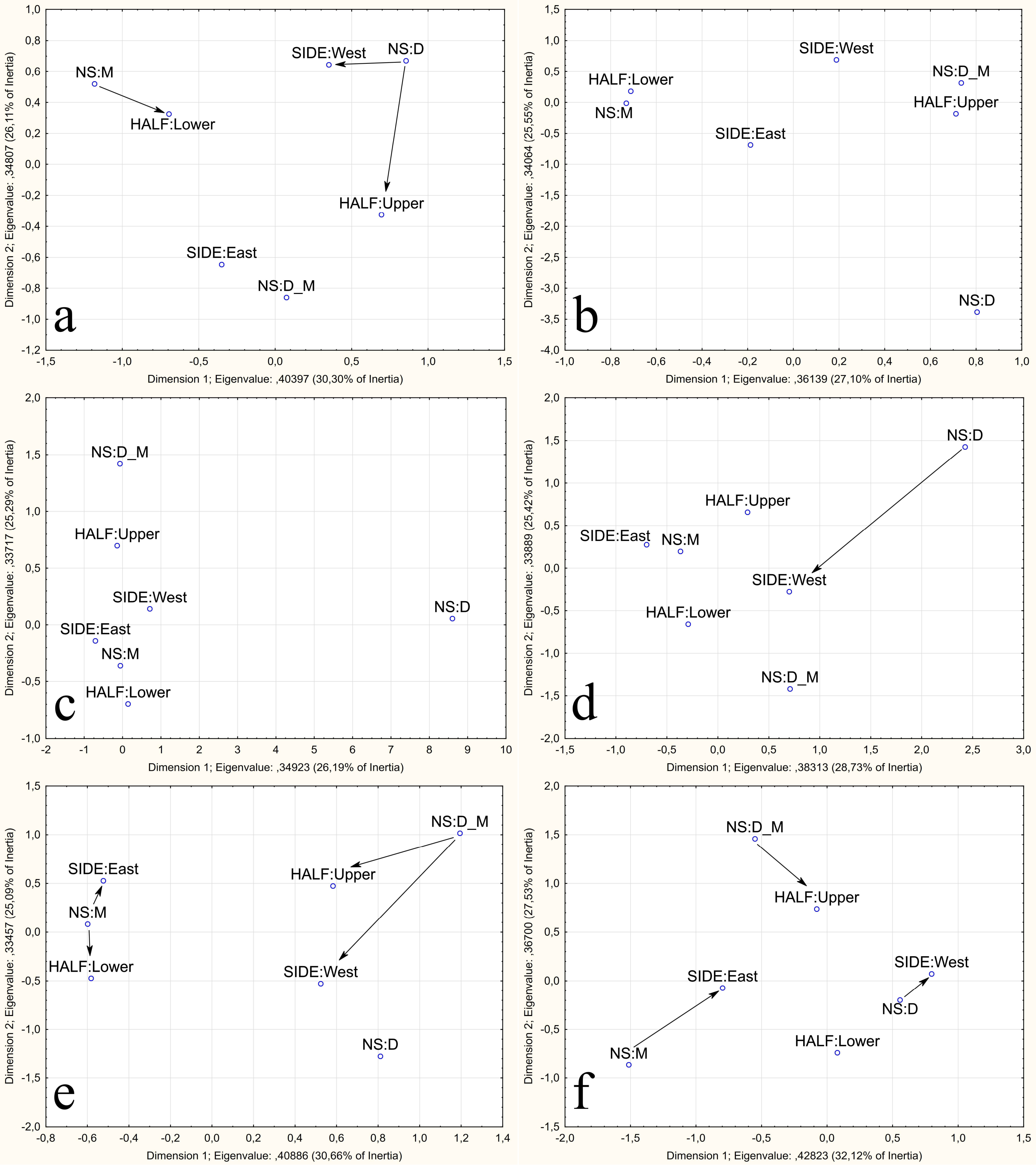

S2 Fig. Distribution of the shoots in the canopy during the evaluation period. Bi-dimensional representation of the MCA (first two dimensions) on the association between different levels of the categorical variables. Side of the plant: west or east; sampling position of the canopy: upper or lower half; stage of new shoots (NS): developing (D), developing-maturing (D_M), or completely mature (M), for each assessment date (a, 02/03/16; b, 23/03/16; c, 13/04/16; d, 04/05/16; e, 25/05/16; f, 15/06/16; g, 05/07/16; h, 27/07/16; i, 17/08/16; j, 07/09/16; k, 28/09/16; l: 19/10/16; m: 09/11/16; n: 30/11/16: o: 21/12/16; p:11/01/17; q: 01/02/17; r: 21/02/17).

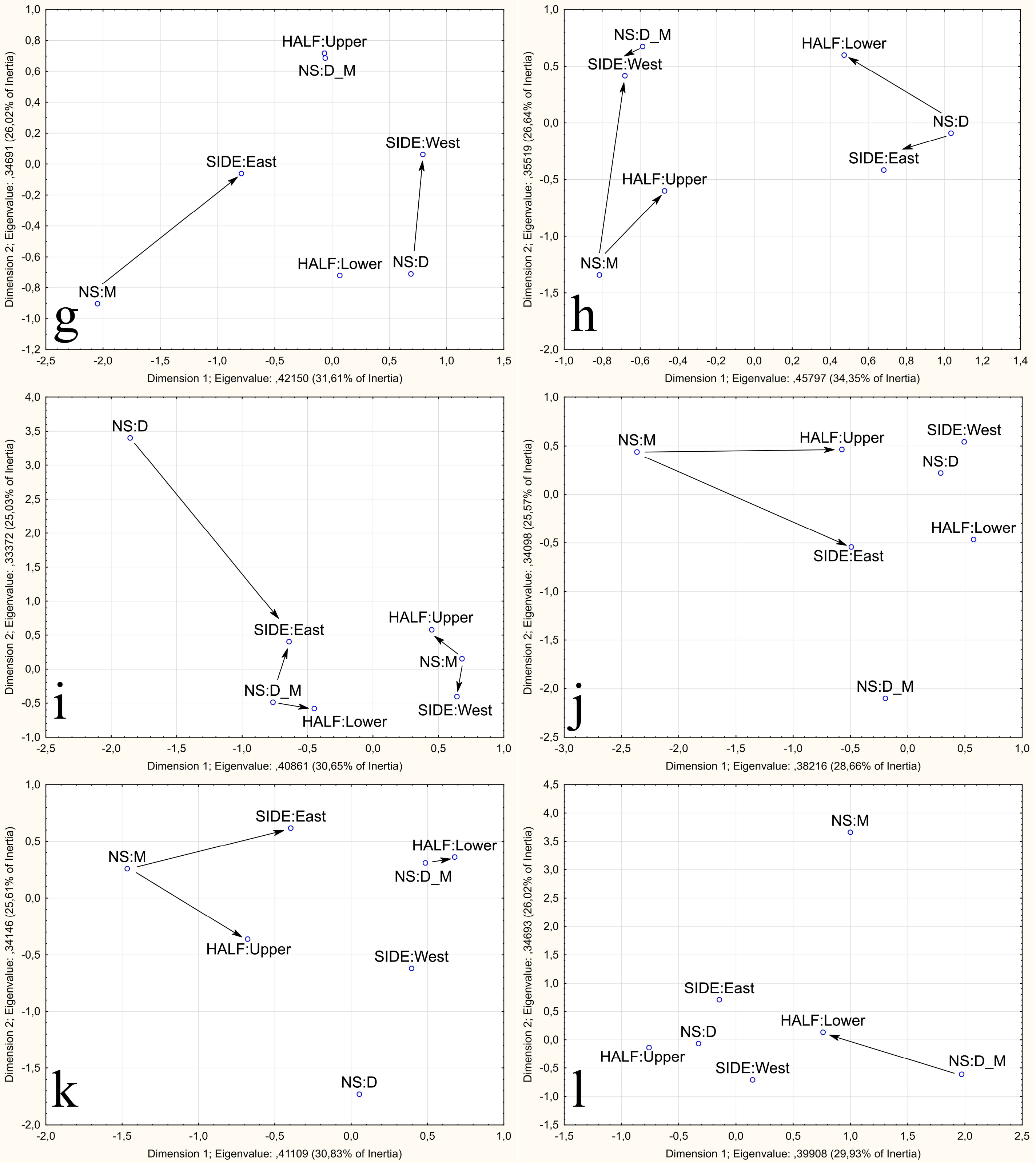

S2 Fig. Distribution of the shoots in the canopy during the evaluation period. *Continue…*

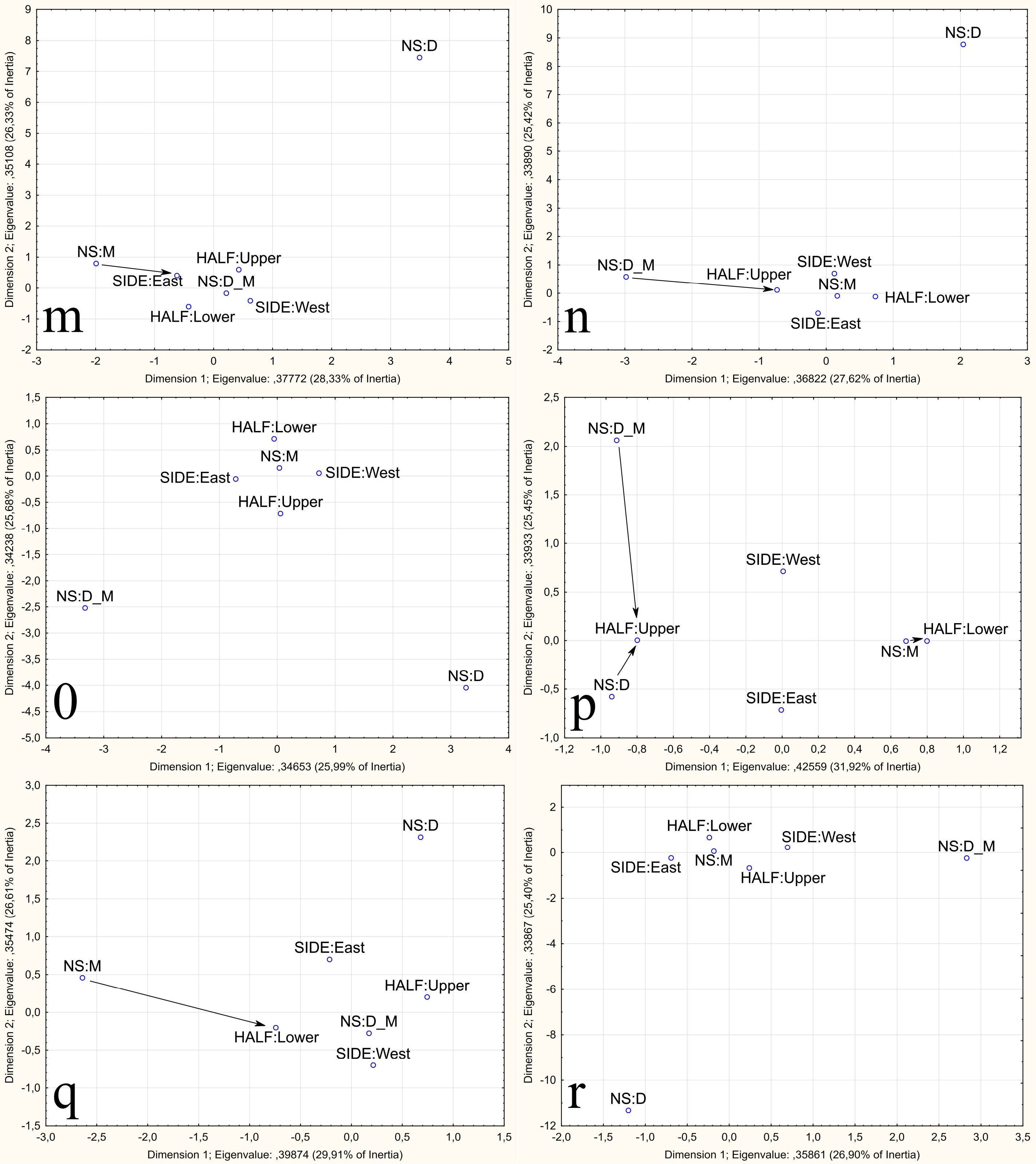

S2 Fig. Distribution of the shoots in the canopy during the evaluation period. *Continue…*
