# Supplementary material for: Optimization of sampling and monitoring of vegetative flushing in citrus orchards
Source: PLoS One. 2020 May 20;15(5):e0233014. doi: 10.1371/journal.pone.0233014 (PMC7239491; doi:10.1371/journal.pone.0233014)
Supplement: S3 Fig — Relationship between the mean number of new shoots inside the projection of the 0.25 m2 square frame in the outer center of the canopy and the maturity index estimated by the predominant flush stage in each sampling position and each side of the canopy (see Materials and Methods for more details). Each point in the plots represents one assessment date and corresponds to the average of 20 sets of randomly selected trees with n = 5 (A, B, C), 10 (D, E, F), 20 (G, H, I), 40 (J, K, L), 80 (M, N, O) trees. The estimated relationship with 160 trees (P, Q, R) is also included. (PDF) [file pone.0233014.s007.pdf]

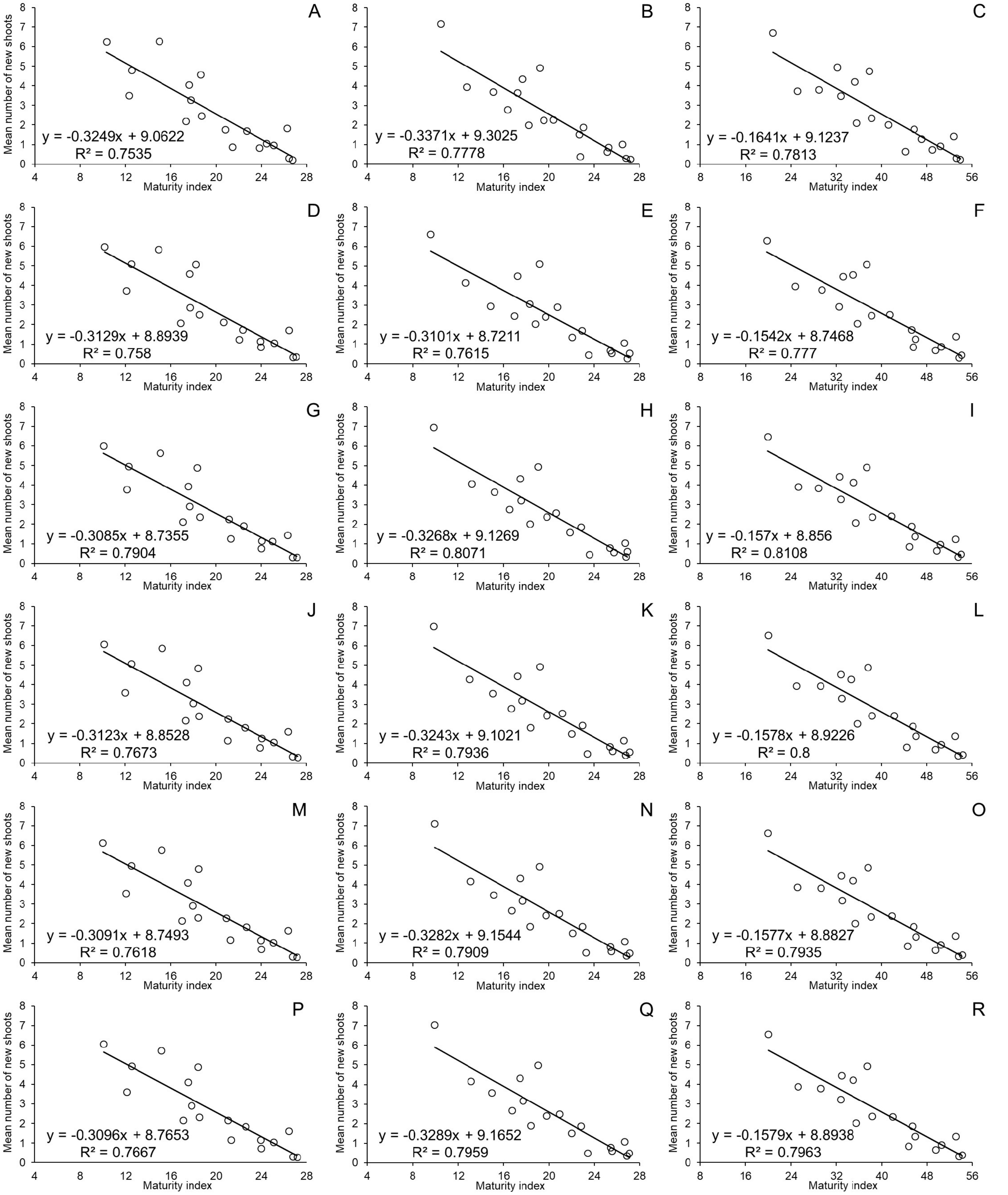

S3 Fig. Overall shoot maturity index. Relationship between the mean number of new shoots inside the projection of the 0.25 m^2 square frame in the outer center of the canopy and the maturity index estimated by the predominant flush stage in each sampling position and each side of the canopy (see Materials and Methods for more details). Each point in the plots represents one assessment date and corresponds to the average of 20 sets of randomly selected trees with n = 5 (A, B, C), 10 (D, E, F), 20 (G, H, I), 40 (J, K, L), 80 (M, N, O) trees. The estimated relationship with 160 trees (P, Q, R) is also included.
